# The Law of Attrition Revisited

**DOI:** 10.2196/jmir.8.3.e20

**Published:** 2006-09-29

**Authors:** Helen Christensen, Andrew Mackinnon

**Affiliations:** ^1^The Centre for Mental Health ResearchThe Australian National UniversityCanberraAustralia

## Eysenbach’s Law of Attrition Revisited

Early last year, Eysenbach published a paper [1] urging the need for a science of attrition. Rather surprisingly, there has not been much debate about the issues raised in the paper (this judged by a Web of Science citation search), despite the clear observation that attrition is a major problem in the use and evaluation of ehealth sites. This letter is an attempt to stimulate more discussion about this important issue.

Eysenbach’s paper gives us three major conceptual advances – the distinction between trial dropout and low/nonusage nondropouts; the proposition of “diffusion of innovation” effectively reversed as a model for the take-up of Internet interventions; and the concept of the “Run in and Withdrawal Design.” The diffusion of innovation reversed is essentially a structural one in that it suggests a number of “systems” features that influence dropout and usage including expectation management, ease of ease of enrolment, ease of dropout, usability, adjunct personal contact, financial commitment, workload, competing events, and experience.

## User Characteristics and Preferences are Important

A number of issues arise from Eysenbach’s proposal. First, the structural or systems model factors in the model may need to be supplemented by consideration of user characteristics. For example, the use and uptake of Web sites in mental health are likely to be modulated by the severity of the user’s mental health problem [2], the users need for anonymity (possibly arising from stigma), lack of alternative resources due to living in a remote location, and the preferences an individual might have for certain sorts of help. The potential impact of these factors in contributing to site adherence is something that needs to be recognized and, more than that, actually studied! There are a number of methods, which although indirect, can provide possible clues for further analysis. These include techniques such as correlating or predicting user characteristics with usage patterns and outcome measures.

A second attribute of users that warrants incorporation in any model of nonusage is an understanding of the expectations that people bring to a Web site, and what they mean by their intention-to-use. For example, many young people do not recognize “lousy feelings” as depression or anxiety, but a brief visit to a Web site provides a “mini-diagnosis” and a label. For them, one module may well fulfill their needs: They have no expectation that they are lining up for a full set of modules. Recognizing these multiple paths and trajectories of web usage means that low usage and dropout do not necessarily coincide with “failure”. Dropouts may well be e-attainers [3].

The multiple uses made of Web sites by different users raises the distinct, but highly relevant issue of the suitability of the Internet to provide full treatment packages for different conditions. The Internet has the capacity to reach many individuals who may never seek formal treatment for mental health services. However, it may well be that the primary role of the Internet in disease prevention will be in the delivery of short positive health messages, rather than the delivery of ‘therapy’ that requires hours of online work. Web site adherence or “stickiness” may cease to be an issue for online sites like MoodGYM when shorter interventions can be demonstrated to lead to similar health outcomes and brief bursts of information lead to increased help-seeking.

## An Example

**Figure 1:**
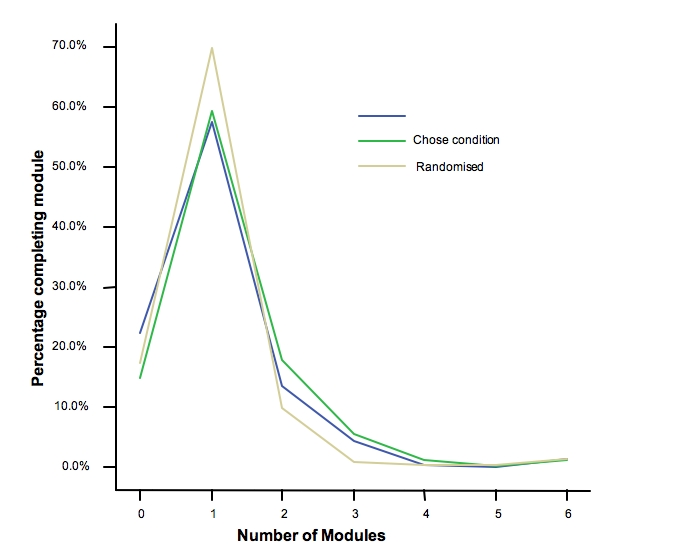
Completion rates of MoodGYM modules as a function of group membership

The following is an example of how attrition may be influenced by the personal characteristics or the preferences of the online users. We are currently conducting a trial of MoodGYM in which those intending to use the program: (a) report that they have been asked to do the trial as a part of their clinician’s treatment plan; (b) chose to do five modules when offered the opportunity to do only fewer than five in the early part of MoodGYM; or (c) are randomized to the MoodGYM condition as a function of an ongoing trial. Figure 1 shows the completion rates of modules as a function of group membership. It is emerging that those who commit to undertake five modules do have a higher likelihood continuing to use the site, although attrition after the third module is almost complete in all groups.

## Similarities and Differences with Clinical Trials

We suggest that Eysenbach’s argument asserting the differences between e-health and traditional clinical trials might be slightly overstated. Many researchers who have been involved with traditional randomized controlled trials (RCTs) of pharmacological treatments in psychiatry will recognize Eysenbach’s characterization of attrition in such settings as extremely optimistic. The dropout of a third of recruited patients in such trials is common with rates exceeding 80% being observed in some long term trials aimed at relapse prevention. Determining whether patients have complied with medication regimes is difficult. In many respects, e-health is in a far stronger position than other studies to detail the low usage of the interventions, given the tracking of length and number of visits to the application. Moreover, e-health interventions have high fidelity: the exact same intervention is potentially available to all the participants.

There are other minor points that need to be made. For example, Eysenbach makes a distinction between users lost to dropout and low usage nondropouts. This model suggests that people discontinue innovations because they are disenchanted or because they seek a better alternative to meet their needs. On reflection there are four theoretically possible usage curves: (i) dropout, low or no usage; (ii) nondropout, low or no usage; (iii) dropout, high usage; and (iv) nondropout, high usage. The dropout, high usage is a person who prefers not to engage with a Web site but undertakes the program under a new user name each visit (if the application is an open web-based one).

## Emerging Statistical Techniques

Up until recently, interventions and clinical trials have been analyzed using classical analysis of variance methods. For these techniques, missing observations arising from participant dropout are a just nuisance factor which is addressed, *a priori*, by admonitions to minimize dropout [4] and, *post hoc*, by analysis of only those participants with complete data or by simplistic and often inappropriate methods of imputation. Mixed or random coefficient models are more recently developed methods that overcome problems due to missing data. These models operate under the assumption that the cause of dropout is measured as part of the available data rather than being contingent on the missing information itself (the missing at random assumption)[5]. These methods enable estimation of the effect of an intervention under the intention to treat model.

Mixed models themselves throw little light on the nature of attrition, its causes or consequences. However, more advanced techniques, based on latent variable modeling, may help us understand the complexity of the multiplicity of paths through and of out interventions. The complier average causal (CACE) model is specially aimed at estimating the effect of an intervention in the presence of noncompliance [6]. Related techniques can be used to empirically delineate classes of response trajectories through and after an intervention [7]. These methods appear to be amenable to extension to accommodate attrition and to model causes of dropout.

## Beyond Attrition

Developing a metric of the attrition attributable to an internet intervention site is an attractive initiative. It would parallel the notion of the acceptability or tolerability of conventional treatments. This concept, usually measured informally or only crudely, recognizes that some treatments, while efficacious, are possibly so odious as to be persevered with by only a few patients who might benefit from them. There are substantial hurdles to such measurements. It will prove difficult, if not impossible to disentangle attrition due to site effects from attrition due to the characteristics of users and the paths they take to a site. More important, to focus exclusive on attrition is to focus on the negative side of e-health interventions. E-health interventions have enormous potential to reach those warranting assistance and to address their needs. Recognizing the fact of high attrition, we need to respond with a science (and an art) of participation and encouragement.
